# Supplementary material for: Limited Impact of Delta Variant’s Mutations on the Effectiveness of Neutralization Conferred by Natural Infection or COVID-19 Vaccines in a Latino Population
Source: Viruses. 2021 Nov 30;13(12):2405. doi: 10.3390/v13122405 (PMC8707683; doi:10.3390/v13122405)
Supplement: Supplementary file 1 [file viruses-13-02405-s001.zip › Supplementary Table S1.pdf]

Supplementary Table S1. Time Between Sample and Vaccination of Healthy and Naturally-Infected Volunteers

HEALTHY VACCINATED VOLUNTEERS				
Numeric ID	Timepoint	Time Between First Dose and 1st Sample Post Vac	Time Between Second Dose and Sample Post 2nd Vac	Time Between First Dose and Last Sample
479	Baseline			
	First Dose			
479.2	Sample P1V	26	16	43
	Second Dose			
479.3	Sample P2V			
112	Baseline			
	First Dose			
112.2	Sample P1V	21	14	42
	Second Dose			
112.3	Sample P2V			
2	Baseline			
	First Dose			
2.2	Sample P1V	13	15	36
	Second Dose			
2.3	Sample P2V			
3	Baseline			
	First Dose			
3.2	Sample P1V	13	15	36
	Second Dose			
3.3	Sample P2V			
243	Baseline			
	First Dose			
243.2	Sample P1V	15	15	36
	Second Dose			
243.3	Sample P2V			
258	Baseline			
	First Dose			
258.2	Sample P1V	18	22	43
	Second Dose			
258.3	Sample P2V			
119	Baseline			
	First Dose			
119.2	Sample P1V	18	18	39
	Second Dose			
119.3	Sample P2V			
190	Baseline			
	First Dose			
190.2	Sample P1V	12	23	44
	Second Dose			
190.3	Sample P2V			
453	Baseline			
	First Dose			
453.2	Sample P1V	23	19	42
	Second Dose			
453.3	Sample P2V			
6	Baseline			
	First Dose			
6.2	Sample P1V	16	14	35
	Second Dose			
6.3	Sample P2V			
383	Baseline			
	First Dose			
383.2	Sample P1V	23	17	40
	Second Dose			
383.3	Sample P2V			
450	Baseline			
	First Dose			
450.2	Sample P1V	21	19	40
	Second Dose			
450.3	Sample P2V			
110	Baseline			
	First Dose			
110.2	Sample P1V	21	19	40
	Second Dose			
110.3	Sample P2V			
480	Baseline			
	First Dose			
480.2	Sample P1V	21	19	40
	Second Dose			
480.3	Sample P2V			
10	Baseline			
	First Dose			
10.2	Sample P1V	14	7	28
	Second Dose			
10.3	Sample P2V			
116	Baseline			
	First Dose			
116.2	Sample P1V	14	7	28
	Second Dose			
116.3	Sample P2V			
380	Baseline			
	First Dose			
380.2	Sample P1V	14	7	28
	Second Dose			
380.3	Sample P2V			
8	Baseline			
	First Dose			
8.2	Sample P1V	14	7	28
	Second Dose			
8.3	Sample P2V			
117	Baseline			
	First Dose			
117.2	Sample P1V	12	6	27
	Second Dose			
117.3	Sample P2V			
254	Baseline			
	First Dose			
254.2	Sample P1V	12	10	31
	Second Dose			
254.3	Sample P2V			
513	Baseline			
	First Dose			
513.2	Sample P1V	19	7	35
	Second Dose			
513.3	Sample P2V			
AVERAGE Days		17.1	14.1	36.2
Naturally-Infected Vaccinated Volunteers				
Numeric ID	Timepoint	Time Between First Dose and 1st Sample Post Vac	Time Between Second Dose and Sample Post 2nd Vac	Time Between First Dose and Last Sample
384.2	Baseline			
	First Dose			
384.3	Sample P1V	16	23	44
	Second Dose			
384.4	Sample P2V			
367.2	Baseline			
	First Dose			
367.3	Sample P1V	14	20	48
	Second Dose			
367.4	Sample P2V			
218	Baseline			
	First Dose			
218.3	Sample P1V	28	21	49
	Second Dose			
218.4	Sample P2V			
376.2	Baseline			
	First Dose			
376.3	Sample P1V	19	21	49
	Second Dose			
376.4	Sample P2V			
313.3	Baseline			
	First Dose			
313.4	Sample P1V	17	16	37
	Second Dose			
313.5	Sample P2V			
382.3	Baseline			
	First Dose			
382.4	Sample P1V	12	19	40
	Second Dose			
382.5	Sample P2V			
511	Baseline			
	First Dose			
511.2	Sample P1V	19	26	47
	Second Dose			
511.3	Sample P2V			
512	Baseline			
	First Dose			
512.2	Sample P1V	19	26	47
	Second Dose			
512.3	Sample P2V			
294.2	Baseline			
	First Dose			
294.3	Sample P1V	26	32	60
	Second Dose			
294.4	Sample P2V			
297	Baseline			
	First Dose			
297.2	Sample P1V	19	17	45
	Second Dose			
297.3	Sample P2V			
AVERAGE Days		18.9	22.1	46.6
