# Supplementary material for: Limited Impact of Delta Variant’s Mutations on the Effectiveness of Neutralization Conferred by Natural Infection or COVID-19 Vaccines in a Latino Population
Source: Viruses. 2021 Nov 30;13(12):2405. doi: 10.3390/v13122405 (PMC8707683; doi:10.3390/v13122405)
Supplement: Supplementary file 1 [file viruses-13-02405-s001.zip › Supplementary Table S2.pdf]

Supplementary Table S2. Time Between Diagnosis and Vaccines for Pre-exposed Individuals

ID	Time (days) between Dx and first vaccine dose	Months
384	111	3.7
367.7	90	3.0
218	85	2.8
376	176	5.9
313	169	5.6
382	148	4.9
511	67	2.2
512	67	2.2
294	201	6.7
297	310	10.3
**Average**	**142.4**	**4.7**

Dx= Diagnosis
