# Supplementary material for: Limited Impact of Delta Variant’s Mutations on the Effectiveness of Neutralization Conferred by Natural Infection or COVID-19 Vaccines in a Latino Population
Source: Viruses. 2021 Nov 30;13(12):2405. doi: 10.3390/v13122405 (PMC8707683; doi:10.3390/v13122405)
Supplement: Supplementary file 1 [file viruses-13-02405-s001.zip › Supplementary Table S3.pdf]

Supplementary Table S3. Neutralization Data Against SARS-CoV-2 and Variants for Pre-exposed Individuals

ID	Wild Type SARS-CoV-2			V1= (RBD, N501Y) Alpha			V2 = (RBD, E484K, K417N, N501Y) Beta			V3 = (RBD, E484K, K417T, N501Y) Gamma			V4 = (RBD, L452R) Epsilon			V5= (RBD, E484Q, L452R) Kappa			V6= (RBD, L452R, T478K) Delta		
	Result (% signal inhibition)																				
	Baseline	First Vaccine Dose	Second Vaccine Dose	Baseline	First Vaccine Dose	Second Vaccine Dose	Baseline	First Vaccine Dose	Second Vaccine Dose	Baseline	First Vaccine Dose	Second Vaccine Dose	Baseline	First Vaccine Dose	Second Vaccine Dose	Baseline	First Vaccine Dose	Second Vaccine Dose	Baseline	First Vaccine Dose	Second Vaccine Dose
511	81	98	97	59	98	98	41	96	96	27	96	96	56	98	98	58	97	97	62	98	98
512	46	87	89	25	91	88	16	82	80	5	80	78	31	90	84	19	81	76	45	76	69
218	72	98	97	31	98	98	26	96	96	6	98	98	31	98	98	11	97	98	45	98	98
376	94	98	97	63	98	98	38	96	96	28	98	98	68	98	98	63	97	97	72	98	98
367	92	98	97	56	98	98	40	97	96	38	98	98	66	98	98	64	97	98	76	98	98
294	52	97	98	23	98	98	17	96	96	19	97	97	24	98	98	11	97	97	28	98	98
384	87	98	98	61	98	98	37	96	96	18	98	98	57	98	98	38	97	98	69	98	98
313	97	99	98	87	98	98	81	97	96	75	98	98	88	98	98	87	98	98	92	98	98
382	72	98	97	39	98	98	18	96	96	16	98	98	36	98	98	34	98	98	42	98	98
297	96	97	97	94	98	98	87	99	97	78	98	98	94	98	98	92	97	98	93	98	98

POS ≥ 30% signal inhibition

**V1**= (RBD, N501Y, Avi & His tag)-HRP	U1100GG280-1:Catalog No: Z03595-100; Name: SARS-CoV-2 Spike protein (RBD, N501Y, Avi & His tag)-HRP; Qty: 1; Size: 100ul
**V2** = (RBD, E484K, K417N, N501Y, Avi & His tag)-HRP	U1100GG280-2:Catalog No: Z03596-100; Name: SARS-CoV-2 Spike protein (RBD, E484K, K417N, N501Y, Avi & His tag)-HRP; Qty: 1; Size: 100ul
**V3** = (RBD, E484K, K417T, N501Y, Avi & His Tag)-HRP	U1100GG280-3:Catalog No: Z03601-100; Name: SARS-CoV-2 Spike protein (RBD, E484K, K417T, N501Y, Avi & His Tag)-HRP; Qty: 1; Size: 100ul
**V4** = (RBD, L452R, Avi & His Tag)-HRP	U1100GG280-4:Catalog No: Z03605-100; Name: SARS-CoV-2 Spike protein (RBD, L452R, Avi & His Tag)-HRP; Qty: 1; Size: 100ul
**V5** = (RBD, E484Q, L452R, Avi & His Tag)-HRP	U1100GG280-5:Catalog No: Z03608-100; Name: SARS-CoV-2 Spike protein (RBD, E484Q, L452R, Avi & His Tag)-HRP; Qty: 1; Size: 100ul
**V6** = (RBD, L452R, T478K, Avi & His Tag)-HRP	U1100GG280-6Catalog No: Z03614-100; Name: SARS-CoV-2 Spike protein (RBD, L452R, T478K, Avi & His Tag)-HRP; Qty: 1; Size: 100ul
