# Supplementary material for: Limited Impact of Delta Variant’s Mutations on the Effectiveness of Neutralization Conferred by Natural Infection or COVID-19 Vaccines in a Latino Population
Source: Viruses. 2021 Nov 30;13(12):2405. doi: 10.3390/v13122405 (PMC8707683; doi:10.3390/v13122405)
Supplement: Supplementary file 1 [file viruses-13-02405-s001.zip › Supplementary Table S4.pdf]

Supplementary Table S4. Neutralization Data Against SARS-CoV-2 and Variants for Healthy Vaccinated Individuals

ID	WT SARS-CoV-2			V1= (RBD, N501Y) Alpha			V2 = (RBD, E484K, K417N, N501Y) Beta			V3 = (RBD, E484K, K417T, N501Y) Gamma			V4 = (RBD, L452R) Epsilon			V5= (RBD, E484Q, L452R) India			V6= (RBD, L452R, T478K) Delta		
	Result (% signal inhibition)																				
	Baseline	First Vaccine Dose	Second Vaccine Dose	Baseline	First Vaccine Dose	Second Vaccine Dose	Baseline	First Vaccine Dose	Second Vaccine Dose	Baseline	First Vaccine Dose	Second Vaccine Dose	Baseline	First Vaccine Dose	Second Vaccine Dose	Baseline	First Vaccine Dose	Second Vaccine Dose	Baseline	First Vaccine Dose	Second Vaccine Dose
479	10	46	97	0	26	97	0	0	87	7	31	89	0	33	98	0	42	97	0	43	97
112	19	96	98	0	79	98	0	72	96	0	68	97	0	87	98	0	83	97	0	91	97
2	10	46	98	3	21	95	0	23	90	5	43	90	0	29	97	0	26	95	0	28	96
3	19	49	98	2	37	94	0	14	86	0	22	83	0	50	96	0	25	96	0	45	97
243	22	76	97	0	46	96	0	27	89	6	32	90	2	42	97	0	36	96	0	56	97
258	20	50	97	0	32	94	0	23	73	0	18	81	0	20	94	0	24	93	0	37	95
119	21	29	97	0	5	86	0	0	61	0	3	63	0	8	93	0	1	91	0	17	94
190	23	28	97	0	3	96	0	0	88	4	0	86	0	0	94	0	7	94	0	4	96
453	12	76	97	1	64	93	0	35	83	0	29	84	0	65	94	0	66	94	0	78	96
6	15	65	98	5	67	96	1	45	88	1	31	78	0	36	91	0	41	91	0	58	94
383	4	71	98	2	59	96	0	44	90	4	51	91	0	44	97	0	58	96	0	63	97
450	4	70	95	0	57	84	1	37	75	0	39	72	8	40	84	0	38	82	0	53	89
110	18	44	97	4	36	92	0	29	89	2	15	88	0	27	92	0	28	92	0	38	94
480	4	70	95	0	25	95	11	22	82	0	25	81	0	18	95	2	27	94	0	36	96
10	24	61	97	0	40	96	0	19	89	0	16	85	1	33	96	0	27	96	0	37	97
116	0	66	97	0	46	97	0	39	92	0	37	89	0	46	97	5	40	96	0	50	97
380	25	61	97	0	30	94	0	49	88	3	43	84	0	50	95	1	39	95	0	49	96
8	26	70	97	2	46	92	0	44	87	0	39	85	6	37	95	0	28	95	0	46	96
117	22	55	97	0	21	89	0	31	86	0	18	78	0	36	95	0	45	95	6	41	96
254	18	57	98	2	30	96	0	28	90	0	32	89	13	45	98	0	37	97	0	47	97
513	17	79	97	5	65	98	0	64	97	2	42	97	17	69	98	1	69	98	0	73	98

POS ≥ 30% signal inhibition

**V1**= (RBD, N501Y, Avi & His tag)-HRP	U1100GG280-1:Catalog No: Z03595-100; Name: SARS-CoV-2 Spike protein (RBD, N501Y, Avi & His tag)-HRP; Qty: 1; Size: 100ul
**V2** = (RBD, E484K, K417N, N501Y, Avi & His tag)-HRP	U1100GG280-2:Catalog No: Z03596-100; Name: SARS-CoV-2 Spike protein (RBD, E484K, K417N, N501Y, Avi & His tag)-HRP; Qty: 1; Size: 100ul
**V3** = (RBD, E484K, K417T, N501Y, Avi & His Tag)-HRP	U1100GG280-3:Catalog No: Z03601-100; Name: SARS-CoV-2 Spike protein (RBD, E484K, K417T, N501Y, Avi & His Tag)-HRP; Qty: 1; Size: 100ul
**V4** = (RBD, L452R, Avi & His Tag)-HRP	U1100GG280-4:Catalog No: Z03605-100; Name: SARS-CoV-2 Spike protein (RBD, L452R, Avi & His Tag)-HRP; Qty: 1; Size: 100ul
**V5** = (RBD, E484Q, L452R, Avi & His Tag)-HRP	U1100GG280-5:Catalog No: Z03608-100; Name: SARS-CoV-2 Spike protein (RBD, E484Q, L452R, Avi & His Tag)-HRP; Qty: 1; Size: 100ul
**V6** = (RBD, L452R, T478K, Avi & His Tag)-HRP	U1100GG280-6Catalog No: Z03614-100; Name: SARS-CoV-2 Spike protein (RBD, L452R, T478K, Avi & His Tag)-HRP; Qty: 1; Size: 100ul
